# Supplementary material for: Altered potassium channel distribution and composition in myelinated axons suppresses hyperexcitability following injury
Source: eLife. 2016 Apr 1;5:e12661. doi: 10.7554/eLife.12661 (PMC4841771; doi:10.7554/eLife.12661)
Supplement: Figure 1—source data 1. — DOI: http://dx.doi.org/10.7554/eLife.12661.004 [file elife-12661-fig1-data1.docx]

**Figure 1**

WB

| **Kv1.1** |  |  |
| --- | --- | --- |
|  | Mean | SEM |
| Naïve | 1 | 0.119539397 |
| Neuroma d7 | 0.270681607 | 0.084539848 |
| Neuroma d21 | 0.301624081 | 0.052766252 |

| **Kv1.2** |  |  |
| --- | --- | --- |
|  | Mean | SEM |
| Naïve | 1 | 0.144668111 |
| Neuroma d7 | 0.1246365 | 0.034286334 |
| Neuroma d21 | 0.107348961 | 0.037181389 |

| **Kv1.4** |  |  |
| --- | --- | --- |
|  | Mean | SEM |
| Control | 1 | 0.246733264 |
| Neuroma D7 | 2.282052295 | 0.217021475 |
| Neuroma d21 | 2.27586328 | 0.367862441 |

| **Kv1.6** | |  |
| --- | --- | --- |
|  | Mean | SEM |
| Control | 0.469757993 | 0.186756166 |
| Neuroma D7 | 1.60464685 | 0.272388194 |
| Neuroma D21 | 1.624262335 | 0.296169653 |
