# Supplementary material for: Altered potassium channel distribution and composition in myelinated axons suppresses hyperexcitability following injury
Source: eLife. 2016 Apr 1;5:e12661. doi: 10.7554/eLife.12661 (PMC4841771; doi:10.7554/eLife.12661)
Supplement: Figure 2—source data 1. — DOI: http://dx.doi.org/10.7554/eLife.12661.006 [file elife-12661-fig2-data1.docx]

**Figure 2**

|  | Typical | split | naked | heminode |
| --- | --- | --- | --- | --- |
| Naïve | 91.50326797 | 2.941176471 | 0 | 5.555555556 |
| Neuroma day7 | 47.53401361 | 23.3531746 | 7.219387755 | 21.89342404 |
| Neuroma day21 | 46.09590542 | 13.50720559 | 11.45959159 | 28.9372974 |
