# Supplementary material for: Altered potassium channel distribution and composition in myelinated axons suppresses hyperexcitability following injury
Source: eLife. 2016 Apr 1;5:e12661. doi: 10.7554/eLife.12661 (PMC4841771; doi:10.7554/eLife.12661)
Supplement: Figure 3—source data 1. — DOI: http://dx.doi.org/10.7554/eLife.12661.008 [file elife-12661-fig3-data1.docx]

**Figure 3**

| mean |  | Nav-end Caspr | Nav-start Kv1.2 | difference |  | Nav- end Caspr | Nav- start caspr2 | difference |
| --- | --- | --- | --- | --- | --- | --- | --- | --- |
|  | naïve | 3.8 | 4.2 | 0.563 |  | 3.84 | 4.333333333 | 0.493333333 |
|  | neuroma | 4.2 | 3.3 | -0.9 |  | 4.1 | 3.2 | -0.8 |
|  | proximal to neuroma | 4.5 | 3.4 | -1.1 |  | 4.2 | 3.6 | -0.5 |
|  |  |  |  |  |  |  |  |  |
|  |  |  |  |  |  |  |  |  |
| sem |  | Nav-end Caspr | Nav-start Kv1.2 | difference |  | Nav- end Caspr | Nav- start caspr2 | difference |
|  | naïve | 0.2 | 0.2 | 0.0891 |  | 0.360061022 | 0.323757142 | 0.092427968 |
|  | neuroma | 0.1 | 0.2 | 0.141 |  | 0.240231606 | 0.261463741 | 0.15974583 |
|  | proximal to neuroma | 0.1 | 0.08 | 0.09 |  | 0.1 | 0.1 | 0.1 |
|  |  |  |  |  |  |  |  |  |
