# Supplementary material for: Altered potassium channel distribution and composition in myelinated axons suppresses hyperexcitability following injury
Source: eLife. 2016 Apr 1;5:e12661. doi: 10.7554/eLife.12661 (PMC4841771; doi:10.7554/eLife.12661)
Supplement: Figure 4—source data 1. — DOI: http://dx.doi.org/10.7554/eLife.12661.010 [file elife-12661-fig4-data1.docx]

**Figure 4**

|  | interloop gap | | axo sc gap |
| --- | --- | --- | --- |
|  | max | min |  |
| Control | 12.59 ±1 | 7.8±0.6 | 9.7±0.4 |
| Neuroma d21 | 18.9 ±1.4 | 7.6±0.7 | 11.4±0.7 |

|  | detached | everted |
| --- | --- | --- |
| Control | 10± 6 | 5±5 |
| Neuroma d21 | 5.5±5 | 12±10 |

| Axonal diameter at the node | | |
| --- | --- | --- |
|  | Control | Neuroma d21 |
| mean | 1.300565476 | 1.530595238 |
| sem | 0.132950889 | 0.199427003 |
