# Supplementary material for: Altered potassium channel distribution and composition in myelinated axons suppresses hyperexcitability following injury
Source: eLife. 2016 Apr 1;5:e12661. doi: 10.7554/eLife.12661 (PMC4841771; doi:10.7554/eLife.12661)
Supplement: Figure 5—source data 1. — DOI: http://dx.doi.org/10.7554/eLife.12661.012 [file elife-12661-fig5-data1.docx]

**Figure 5**

IHC

|  | Control | Neuroma d21 |
| --- | --- | --- |
| mean | 1 | 0.4 |
| sem | 0.04 | 0.02 |

WBs

|  | Control | Neuroma d21 |
| --- | --- | --- |
| mean | 1 | 0.71 |
| sem | 0 | 0.08 |
