# Supplementary material for: Altered potassium channel distribution and composition in myelinated axons suppresses hyperexcitability following injury
Source: eLife. 2016 Apr 1;5:e12661. doi: 10.7554/eLife.12661 (PMC4841771; doi:10.7554/eLife.12661)
Supplement: Figure 6—source data 1. — DOI: http://dx.doi.org/10.7554/eLife.12661.014 [file elife-12661-fig6-data1.docx]

**Figure 6**

**WB quantification**

| **Kv1.1** |  |  |
| --- | --- | --- |
|  | Mean | SEM |
| Con | 1 | 0.218395301 |
| N d 7 | 0.636594167 | 0.171278172 |
| N d21 | 0.654998035 | 0.18191691 |

| **Kv1.2** |  |  |
| --- | --- | --- |
|  | Mean | SEM |
| Con | 1 | 0.191335271 |
| N d 7 | 0.366838698 | 0.112821838 |
| N d21 | 0.321859499 | 0.085074192 |

| **Kv1.4** |  |  |
| --- | --- | --- |
|  | Mean | SEM |
| Naïve | 1 | 0.205047053 |
| N d7 | 0.926948399 | 0.259703793 |
| N d21 | 0.783132212 | 0.176190549 |

| **Kv1.6** |  |  |
| --- | --- | --- |
|  | Mean | SEM |
| Naïve | 1 | 0.210626544 |
| N d7 | 1.058176246 | 0.206662185 |
| N d21 | 0.862388512 | 0.131684913 |
