# Supplementary material for: Altered potassium channel distribution and composition in myelinated axons suppresses hyperexcitability following injury
Source: eLife. 2016 Apr 1;5:e12661. doi: 10.7554/eLife.12661 (PMC4841771; doi:10.7554/eLife.12661)
Supplement: Figure 7—source data 1. — DOI: http://dx.doi.org/10.7554/eLife.12661.016 [file elife-12661-fig7-data1.docx]

**Figure 7**

IHC

|  | Control | Neuroma |
| --- | --- | --- |
| Kv1.2 | 90±10 | 13.3±8.16 |
| Kv1.4 | 0±0 | 92.5±7.4 |
| Kv1.6 | 0±0 | 73.5±8.8 |

WB

|  | Control | Neuroma |
| --- | --- | --- |
|  |  |  |
| Kv1.2 | 1±0.2 | 0.1±0.03 |
| Kv1.4 | 1±0.3 | 8.8±3 |
| Kv1.6 | 1±0.3 | 12.1±5.7 |
