# Supplementary material for: Altered potassium channel distribution and composition in myelinated axons suppresses hyperexcitability following injury
Source: eLife. 2016 Apr 1;5:e12661. doi: 10.7554/eLife.12661 (PMC4841771; doi:10.7554/eLife.12661)
Supplement: Figure 8—source data 1. — DOI: http://dx.doi.org/10.7554/eLife.12661.018 [file elife-12661-fig8-data1.docx]

**Figure 8**

|  |  | Kv 1.2 |  |  |  | Kv 1.4 |  |  |  | Kv 1.6 |  |  |  | caspr2 |  |  |
| --- | --- | --- | --- | --- | --- | --- | --- | --- | --- | --- | --- | --- | --- | --- | --- | --- |
|  |  | Nav-end Caspr | Nav-start Kv1.2 | difference . | | Nav- end caspr | Nav- start Kv1.4 | difference . | | Nav- end caspr | Nav- start Kv1.6 | difference . | | Nav- end caspr | Nav- start caspr2 | difference . |
|  | naïve | 3.594230769 | 4.12 | 0.525769231 |  |  |  |  |  |  |  |  |  | 3.8 | 4.3 | 0.5 |
|  | SNT d21 | 3.970526316 | 2.505789474 | -1.464736842 |  | 3.6506 | 1.94845 | -1.70215 |  | 3.4 | 1.6419375 | -1.077225 |  | 3.4 | 2.52 | -0.88 |
|  |  |  |  |  |  |  |  |  |  |  |  |  |  |  |  |  |
|  | SEM |  |  |  |  |  |  |  |  |  |  |  |  |  |  |  |
|  |  | Nav-Caspr | Nav-Kv1.2 | difference |  |  |  |  |  |  |  |  |  |  |  |  |
|  | naïve | 0.310889133 | 0.338567116 | 0.088225538 |  |  |  |  |  |  |  |  |  | 0.3 | 0.4 | 0.2 |
|  | SNT d21 | 0.419618647 | 0.470260794 | 0.325516565 |  | 0.462551285 | 0.395407447 | 0.601405983 |  | 0.478600459 | 0.465986003 | 0.262654331 |  | 0.3 | 0.3 | 0.1 |
|  |  |  |  |  |  |  |  |  |  |  |  |  |  |  |  |  |
