# Supplementary material for: Altered potassium channel distribution and composition in myelinated axons suppresses hyperexcitability following injury
Source: eLife. 2016 Apr 1;5:e12661. doi: 10.7554/eLife.12661 (PMC4841771; doi:10.7554/eLife.12661)
Supplement: Figure 9—source data 1. — DOI: http://dx.doi.org/10.7554/eLife.12661.020 [file elife-12661-fig9-data1.docx]

**Figure 9**

Spontaneous Activity (panel A)

|  | **PRE TOXIN** | **NEUROMA TOXIN** | **DRG TOXIN** |
| --- | --- | --- | --- |
| **NAÏVE** | 10/222 | 12/222 | 21/222 |
| **DAY 2** | 64/291 | 76/291 | 58/291 |
| **DAY 7** | 15/241 | 27/241 | 33/212 (lower total due to death of 1 animal before toxin application on DRG) |
| **DAY 21** | 6/237 | 17/237 | 42/237 |

Mechanosensitivity (panel B)

|  | **PRE TOXIN** | **NEUROMA TOXIN** |
| --- | --- | --- |
| **4 g** | 46/259 | 55/259 |
| **8 g** | 57/259 | 66/259 |
| **15 g** | 83/366 | 109/366 |
