# Supplementary material for: Altered potassium channel distribution and composition in myelinated axons suppresses hyperexcitability following injury
Source: eLife. 2016 Apr 1;5:e12661. doi: 10.7554/eLife.12661 (PMC4841771; doi:10.7554/eLife.12661)
Supplement: Figure 10—source data 1. — DOI: http://dx.doi.org/10.7554/eLife.12661.022 [file elife-12661-fig10-data1.docx]

**Figure 10**

**A**

| Neuroma day 7 | |  |
| --- | --- | --- |
|  | saline | DTX |
| pre-injection | 12.2±0.9 | 13.4±0.8 |
| post-injection | 12.46±1 | 6.9±0.9 |
|  |  |  |
| Neuroma day 21 | |  |
|  | saline | DTX |
| pre-injection | 47±1.1 | 42.8±2.6 |
| post-injection | 49.7±2.4 | 9.8±2.1 |

**B**

|  | Mean |  |  | SEM |  |
| --- | --- | --- | --- | --- | --- |
|  | Saline | CP339818 |  | Saline | CP339818 |
| BL | 133.2175193 | 164.3002759 |  | 21.54844908 | 38.01210745 |
| N D7 | 41.80750835 | 39.3204509 |  | 3.61355395 | 4.353067155 |
| N d21 | 73.06476587 | 91.2072701 |  | 13.89562566 | 4.04163694 |
